# Circulating Tumor Cells Detection and Counting in Uveal Melanomas by a Filtration-Based Method

**DOI:** 10.3390/cancers6010323

**Published:** 2014-02-07

**Authors:** Cinzia Mazzini, Pamela Pinzani, Francesca Salvianti, Cristian Scatena, Milena Paglierani, Francesca Ucci, Mario Pazzagli, Daniela Massi

**Affiliations:** 1 Department of Translational Medicine and Surgery, Università di Firenze, Firenze 50134, Italy; E-Mails: cinzia.mazzini@unifi.it (C.M.); cristian.scatena@email.it (C.S.); mile.paglierani@gmail.com (M.P.); francescaucci@gmail.com (F.U.); daniela.massi@unifi.it (D.M.); 2 Department of Biomedical, Experimental and Clinical Sciences, Università di Firenze, Firenze 50139, Italy; E-Mails: francesca.salvianti@unifi.it (F.S.); m.pazzagli@dfc.unifi.it (M.P.)

**Keywords:** CTC, circulating tumor microemboli (CTM), tumor marker, liquid biopsy, ISET

## Abstract

Uveal melanoma is one of the most deadly diseases in ophthalmology for which markers able to predict the appearance of metastasis are needed. The study investigates the role of circulating tumor cells (CTC) as a prognostic factor in this disease. We report the detection of circulating tumor cells by Isolation by Size of Epithelial Tumor cells (ISET) in a cohort of 31 uveal melanoma patients: we identified single CTCs or clusters of cells in 17 patients, while the control population, subjects with choroidal nevi, showed no CTC in peripheral blood. The presence of CTCs did not correlate with any clinical and pathological parameter, such as tumor larger basal diameter (LBD), tumor height and TNM. By stratifying patients in groups on the basis of the number of CTC (lower or higher than 10 CTC per 10 mL blood) and the presence of CTC clusters we found a significant difference in LBD (*p* = 0.019), Tumor height (*p* = 0.048), disease-free and overall survival (*p* < 0.05). In conclusion, we confirm the role of CTC as a negative prognostic marker in uveal melanoma patients after a long follow-up period. Further characterization of CTC will help understanding uveal melanoma metastasization and improve patient management.

## 1. Introduction

Uveal melanoma is the most common primary tumor of the eye and is one of the most deadly diseases in ophthalmology [1]. It can affect any part of the uveal tract, but choroidal melanoma is predominant (86.3%), while iris and ciliary body melanomas are far less frequent [2]. 

Despite dramatic improvement in the diagnosis and treatment of the primary tumor the survival rate of uveal melanoma patients has not improved in the last half century. Numerous clinical and histopathological features have been investigated in order to predict uveal melanoma prognosis. Size of tumor is one of the most important clinical features for predicting prognosis of uveal melanoma. Increasing tumor thickness, as well as increasing largest basal tumor diameter carries increased risk for metastases [3].

Notwithstanding the improvement in characterization of the primary tumor, an effective tumor marker able to predict the appearance of metastatic disease is still not available [4]. The most likely explanation for this is that distant metastases result from tumor cells dissemination through the blood stream even before the disease becomes clinically evident [5]. 

For this reason the investigation of circulating tumor cells (CTC), which might have the potential to colonize certain organs and to give rise to clinically manifest metastatic disease, would be of great importance for the understanding of the metastatic process [6].

The detection of CTC is particularly cumbersome and technically demanding, but it would be of great help to achieve early diagnosis, as well as to evaluate the risk of development of distant metastases and to predict relapse [7].

Several methods are available to isolate and count CTC in the bloodstream. Some of them directly detect the tumor cells present into the circulation, while others rely on the detection of particular tumor-specific biomarkers to indirectly detect tumor cell presence. 

In a previous paper [8] we studied a group of 41 patients affected by uveal melanoma including small, medium and large size tumors (Collaborative Ocular Melanoma [9]) followed for a period of up to 55 months. The study was performed by RT-qPCR for the measurement of tyrosinase mRNA in blood as a means to detect and indirectly quantify the number of CTC. On the other hand methods aimed at isolating circulating tumor cells from blood samples can provide direct evidence of tumor cell presence in blood and allow cell counting and characterization. To reach this object it is possible to choose among a variety of different assays relying on several principles. Those based on filtration have the advantage of avoiding cell selection due to the immunomagnetic capture procedures often included in assay methods for CTC detection. The use of a filtration procedure allows CTC detection based on the bigger dimension of tumor-deriving cells in respect to white blood cells. The technique is easy to perform and doesn’t require expensive equipment. On the other hand, extensive and qualified work is required to verify the presence of CTC on filters and for their further immunohistochemical characterization, mainly assigned to an expert anatomo-pathologist. 

The object of the present paper is the detection of circulating tumor cells by Isolation by Size of Epithelial Tumor cells (ISET) in a larger cohort of uveal melanoma patients. The results were correlated to the main clinical parameters and to the survival of the tumor patients.

## 2. Results and Discussion

### 2.1. Detection of Circulating Uveal Melanoma Cells by ISET

CTC identification was achieved by morphological analysis. As previously reported [10], morphological criteria for the identification of circulating melanoma cells included: (i) cell size ≥16 µm, (ii) nucleo-cytoplasmic ratio ≥50%, (iii) irregular nuclear shape, (iv) hyperchromatic nucleus, and (v) basophilic cytoplasm. Leukocytes were also retained on the membrane in a low percentage, often trapped within the pore lumina. Such cells were easily identified due to their smaller size and peculiar nuclear morphology. Superposition of cells or cells and pores, nude nuclei, or excess of staining were regarded as artifacts.

**Figure 1:**
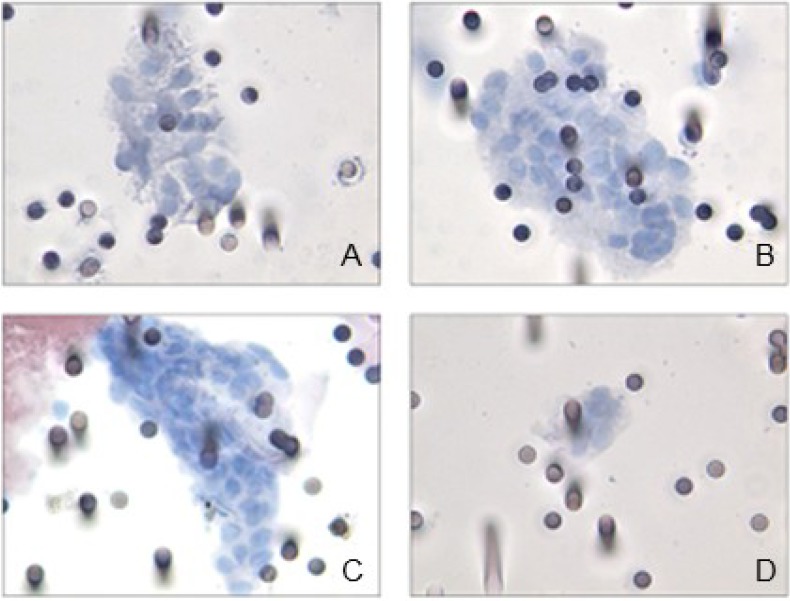
ISET-positive samples display microemboli (**A**–**C**) and isolated melanoma cells (**D**) on filters stained with haematoxylin and eosin.

CTC were not detected in the control group represented by ten subjects showing ocular lesion related to choroidal nevi. Likewise, in 14/31 patients we did not indentify any CTC, while 17/31 (55%) subjects showed CTC on filters (Figure 1). In a subgroup of CTC-positive patients (8/17 corresponding to 47%), tumor cells were observed both in single units and in microemboli or clusters of cells (CTM) (Figure 1). 

**Figure 2:**
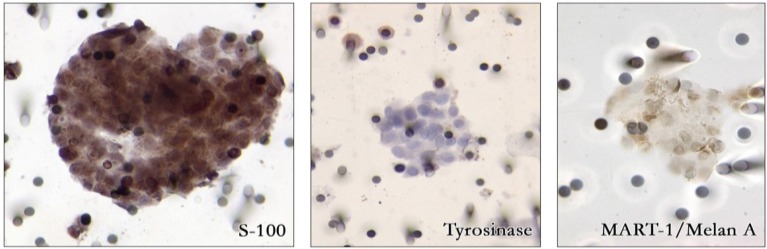
Immunohistochemical analysis of circulating tumor microemboli with S-100, tyrosinase and Mart-1/Melan A antibodies identifying circulating melanoma cells.

For immunohistochemical characterization, CTC isolated on filter were submitted to analysis with anti-S100 protein, MART-1, or tyrosinase antibody identifying circulating melanoma cells as weakly (tyrosinase) to moderately (MART-1) and strongly (S100) stained cells (Figure 2). In the nine patients without CTM, CTC number varied from 2 to 50 CTC/10 mL blood with a median value of 8 CTC/10 mL blood. The presence of CTM prevented the pathologist from performing an accurate counting of the cells circulating in the blood.

### 2.2. Correlation of CTC Number and CTM with Tumor Characteristics

Considering patients for the presence or absence of CTC in peripheral blood, no significant correlation was shown between CTC positivity and the main clinico-pathological parameters, including tumor larger basal diameter (LBD), tumor height and TNM (see Table 1).

**Table 1:** Case study characteristics and their association with CTC presence in uveal melanomas. This table reports the number of CTC-positive and CTC-negative cases according to patients’ clinico-pathological prognostic parameters.

Parameters	All patients (n = 31)	CTC Positive (n = 17)	CTC Negative (n = 14)	*p* value CTC^+^ *vs.* CTC^−^
Age (years)				
median	72	72	74	0.564 ^a^
min-max	52–89	52–82	54–89	
Sex				
Female	14	7	7	0.725 ^b^
Male	17	10	7	
LBD (mm)				
median	10.0	9.8	9.7	0.691 ^a^
min-max	3.0–23.4	3.0–23.4	5.6–20.0	
<14.0 mm	24	12	12	0.412 ^b^
>14.0 mm	7	5	2	
Tumor Height (mm)				
median	4.2	3.5	4.35	0.796 ^a^
min-max	1.8–13.5	1.8–13.0	2.2–13.5	
<7.0 mm	23	12	11	0.698 ^b^
>7.0 mm	8	5	3	
TNM				
T1N0M0	7	4	3	0.557 ^b^
T2N0M0	17	8	9	
T3N0M0	4	3	1	
T4N0M0	1	0	1	
T1N0M1	1	1	0	
T4N0M1	1	1	0	

^a^: Mann-Whitney; ^b^: Chi-square test.

Patients’ stratification according to the absolute CTC number identified three categories characterized by CTC numbers lower or higher than 10 CTC/10 mL blood and by the presence of microemboli. The adopted cut-off value of for patients’ stratification derives from the analytical procedure which enables the simultaneous filtration of ten 1 mL-aliquots of blood with a reported sensitivity of 1 cell/mL [11]. A statistically significant difference was found in the distribution based on LBD (*p* = 0.019; Table 2) and tumor height (*p* = 0.048; Table 3).

**Figure 3:**
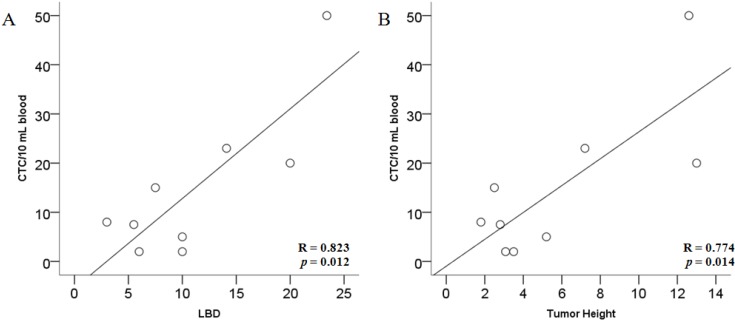
Linear regression analysis between CTC count and lager basal diameter LBD (**A**) and tumor height (**B**).

In the nine patients without CTM, the number of CTC was significantly correlated to LBD (Pearson’s correlation coefficient R = 0.823, *p* = 0.006) and Tumor Height (Pearson’s correlation coefficient R = 0.774, *p* = 0.014) (Figure 3).

**Table 2:** Correlation of CTC count with lager basal diameter LBD.

LBD (mm)	<10 CTC/10 mL blood	>10 CTC/10 mL blood	CTM	Total
<14.0 mm	17	1	6	24
>14.0 mm	2	3	2	7
Total	19	4	8	31

Chi-square test *p* = 0.019.

**Table 3:** Correlation of CTC count with tumor height.

Tumor Height (mm)	<10 CTC/10 mL blood	>10 CTC/10 mL blood	CTM	Total^-^
<7.0 mm	16	1	6	23
>7.0 mm	3	3	2	8
Total	19	4	8	31

Chi-square test *p* = 0.048.

### 2.3. Correlation of CTC Number and CTM with Patients’ Survival

**Figure 4:**
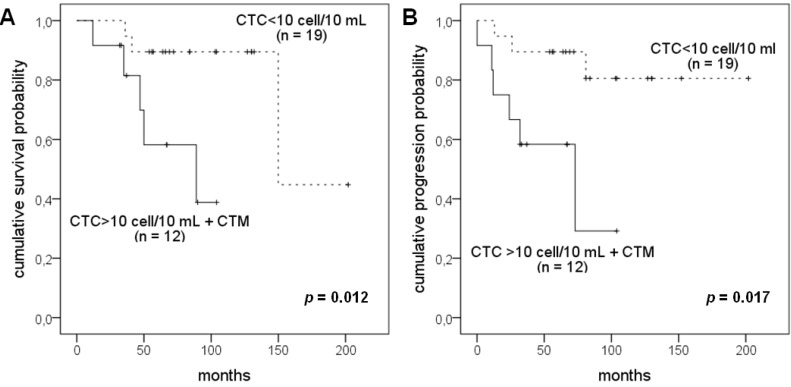
Kaplan-Meier curves for disease free (**A**) and overall survival (**B**) in the whole case study.

The evaluation of the impact of CTC positivity on the survival of the patients with uveal melanoma was performed by univariate analysis. Patients were categorized into two groups: (i) subjects with less than 10 CTC/10 mL of blood; (ii) subjects with more than 10 CTC/10 mL of blood and CTM. Kaplan-Meier curves calculated for the two groups evidence a significantly different disease-free survival (Log Rank test *p* = 0.012) and overall survival (Log Rank test *p* = 0.017) as illustrated in Figure 4. 

### 2.4. Discussion

Herein we report the feasibility of a filtration based method (ISET, isolation by size of epithelial tumor cells) to detect CTC and CTM in uveal melanoma patients. The filtration method was previously employed in our laboratory for CTC detection in breast cancer [12], in cutaneous melanoma patients [10] as well as in patients affected by adrenocortical carcinomas [13] demonstrating to represent a valid approach for CTC detection in any kind of tumor, irrespective of the expression of tumor-specific markers.

In this study CTC analysis was performed in uveal melanomas with a follow up time of several years from the diagnosis and on patients showing ocular lesions related to choroidal nevi, included in the study as the control group. We verified the expected absence of CTC in the control population, while, in our cohort of uveal melanoma patients, we identified 17 CTC-positive subjects with a high percentage (8/17) exhibiting CTC aggregates on filters. As demonstrated by other authors [14], the possibility that CTM might be an artifact caused by sample manipulation can be excluded taking also into account that all clinical specimens were processed identically and CTM were observed only in a subset of samples. CTM are thought to have higher metastatic potential compared to that of isolated CTC [15]. Moreover, the absence of proliferating and of apoptotic cells within CTM supports the hypothesis of a survival advantage for this cells as well as of a theoretical increased chemoresistance in comparison to single proliferating CTC. It is presumable that CTM differ from isolated CTC also in the mechanism of detachment from the primary tumor resulting from a “collective migration” [14].

Similarly to what recently reported by Suesskind and coworkers [6], we could no evidence any significant relationship between CTC presence (CTC-negative versus CTC-positive samples) and patients (sex, age) or tumor characteristics (LBD, Tumor height, TNM). Nevertheless the 84% of patients with less than 10 CTC/10 mL blood showed LBD < 14 mm, while 3/4 patients (75%) with CTC number higher than 10 had larger diameter. The same distribution could be evidenced when analyzing samples on the basis of tumor height. Moreover, a significant correlation between CTC count and both LBD and Tumor height could be evidenced in the nine CTC-positive patients without CTM.

Interestingly, considering the positivity rate calculated on the basis of tumor stage classification, it is evident that ISET could detect a higher rate of CTC positivity in T1 and T2 stages in comparison to Ulmer and coworkers’ results [16] corresponding to an overall lower frequency of CTC detection (see Table 1).

The evaluation of the impact of CTC positivity on the survival of the patients with uveal melanoma showed that CTC-negative patients as well as positive patients with less than 1 CTC per mL of blood showed a better disease free and overall survival than patients with more than 1 CTC per mL of blood, including those demonstrating CTM. Our results confirm the findings of a recent paper [17] reporting a strong association of CTC count with progression-free and overall survival. 

## 3. Experimental

### 3.1. Patients and Sample Collection

Thirty-one patients with uveal melanoma, consecutively examined at the Eye Clinic, Department of Oto-neuro-ophthalmology University of Florence, Italy, were enrolled in the study. Patients’ characteristics are reported in Table 1. The study was approved by the Institutional Review Board and an informed consensus was obtained from all patients.

Peripheral blood samples for the isolation of circulating tumor cells by the ISET method were obtained either at diagnosis, at the time of therapy or at subsequent outpatient visit. All patients were treated for the primary ocular lesion with surgery (enucleation) or radiotherapy (brachitherapy with I^125^ or proton beam therapy). Four patients had been treated with proton beam, at diagnosis, in a different Hospital and submitted to blood sampling after 2–5 years from the diagnosis when they were visited at the Eye Clinic in Florence for the periodic control.

Absence of metastatic melanoma at the time of blood sampling was confirmed by clinical evaluation, routine biochemistry (*i.e.*, liver enzymes, and lactate dehydrogenase levels), and liver ultrasonography in all patients except two. As the control group, 10 patients showing ocular lesion related to choroidal nevi were enrolled. For ISET, 10 mL of blood were collected in EDTA tubes and processed within 4 h. 

### 3.2. Isolation by Size of Epithelial Tumor Cells (ISET)

Blood samples from 31 uveal melanoma patients were collected and submitted to ISET. ISET was carried out using a modification of a previously described [10] module of filtration kindly provided by Metagenex (Paris, France) and a disposable filtration block (ISET Metablock, Metagenex) containing a membrane with calibrated 8-μm diameter pores. The module of filtration has 10 spot-compartments making it possible to load and filter 10 individual samples in parallel. Peripheral blood (8–10 mL) from patients with uveal melanoma was collected on buffered EDTA, diluted 1:10 with the ISET Metabuffer^®^ (Metagenex), incubated for 10 min at room temperature, and filtered. Ten milliliter of diluted solution, corresponding to 1 mL of undiluted blood, was loaded into each compartment and filtered by controlled aspiration under vacuum (0.1 Bar). The membrane was then washed once with phosphate-buffered saline (PBS), disassembled from the filtration module, and allowed to air-dry. The ISET patents for ISET technology are now exclusively licensed to Rarecells (Paris, France).

### 3.3. Cell Staining and Immunostaining

After rehydration with PBS, the spots obtained on the filter, each one corresponding to 1 mL of filtered blood, were stained with haematoxylin and eosin. Hematoxylin S (Merck KGaA, 64271 Darmstadt, Germany) was applied to the membrane for 1 min, followed by 1% eosin Y (Thermo Electron Corporation, Thermo Fisher Scientific Inc., Waltham, MA, USA) for 45 s. 

For immunostaining the whole Metafilter was hydrated with Tris Buffered Saline (TBS, pH 7.4). Having removed the white part of Metafilter, the filter was rinsed with TBS for one minute, the excess of TBS was removed with the absorbing paper and the Metafilter was put on the paraffin film in a humid chamber. Each spot was incubated for 5 min at room temperature with 70 µL of permeabilized buffer: in each immunostaining step, we covered the spots with a coverslip 20 × 20 mm to contain the liquid. The filter was washed quickly in a bath containing distilled water and incubated for 1 h on each spot with 70 µL Polyclonal anti-human S-100 Protein Antibody (Dako, Glostrup, Denmark) diluted 1:100 in Antibody Diluent (Ventana Medical Systems, Tucson, AZ, USA). The Metafilter was washed one time with wash buffer for one minute and put again in a bath containing distilled water. Staining was achieved treating the spots with 70 µL EnVision™ Detection System, Peroxidase/DAB, (Dako) for 40 min at room temperature followed by 3,3'-diaminobenzidine (Dako) for 10 min at room temperature as chromogen. Then the Metafilter has been placed on the horizontal surface (a paraffin film) and the cells nuclei were slightly counterstained with Mayer’s hematoxylin for 6 min. Finally the spots were rinsed with running water, dried for 10 min at room temperature and mounted on slide with Faramount Aqueous mounting medium.

### 3.4. Statistical Analysis

Statistical analysis of the results was carried out using the SPSS software package, release 20.0.1 (SPSS INC, Chicago, IL, USA). Statistical differences between groups were assessed using Chi-square test for discontinuous variables and Mann-Whitney test for the continuous ones. *p* values <0.05 were considered statistically significant. The cumulative survival probability in various subgroups was calculated using the Kaplan-Meier life tables and differences assessed with the log rank method.

## 4. Conclusions

In conclusion, here we confirm the role of CTC as a negative prognostic marker in uveal melanoma patients after a long follow-up period, up to 15 years after diagnosis. Further characterization of CTC, also at the molecular level, will help understanding uveal melanoma metastatic process and improving patients’ management. Other molecular markers can be investigated in the view of a non-invasive monitoring of disease progression in this pathology characterized by long survival time.
